# Effect of home-based isometric handgrip exercise with a commercially available device on blood pressure in older adults with hypertension: A randomized controlled trial

**DOI:** 10.1371/journal.pone.0342563

**Published:** 2026-03-04

**Authors:** Jirapa Champaiboon, Tanya Jitkaew, Sarissa Rangkla, Aisawan Petchlorlian

**Affiliations:** 1 Department of Rehabilitation Medicine, Faculty of Medicine, Chulalongkorn University, Bangkok, Thailand; 2 Department of Rehabilitation Medicine, King Chulalongkorn Memorial Hospital, The Thai Red Cross Society, Bangkok Thailand; 3 Division of Geriatric Medicine, Department of Medicine, Faculty of Medicine, Chulalongkorn University, Bangkok Thailand; 4 Geriatric Excellence Centre, King Chulalongkorn Memorial Hospital, The Thai Red Cross Society, Bangkok Thailand; Japanese Academy of Health and Practice, JAPAN

## Abstract

**Background:**

Isometric handgrip exercise may help reduce blood pressure, but its effectiveness using inexpensive, home-based devices remains unclear. This study aimed to evaluate the impact of home-based isometric handgrip exercise on resting systolic blood pressure (SBP) in hypertensive older adults. Secondary outcomes included diastolic blood pressure (DBP), heart rate (HR), grip strength, safety, and satisfaction.

**Methods:**

A randomized controlled trial was conducted at the Comprehensive Geriatric Clinic, King Chulalongkorn Memorial Hospital, Bangkok, Thailand. Thirty hypertensive participants aged over 60 with handgrip maximum voluntary contraction (MVC) of 10–40 kg were randomized into two groups. The intervention group (n = 15) performed isometric handgrip exercises using spring devices at 20–50% MVC. The control group (n = 15) performed non-resistive handgrip exercises. Both groups exercised 3 times/week for 8 weeks (4 sets of 2-minute holds, 1-minute rest between sets).

**Results:**

The intervention group showed significant reductions in SBP (−7.33 ± 9.63 mmHg, *P* = 0.011) and DBP (−3.6 ± 5.79 mmHg, *P* = 0.03). Grip strength improved in both groups, but only SBP reduction showed a significant between-group difference (−8.19 mmHg, *P* = 0.03). No complications were reported, and satisfaction was high in the intervention group.

**Conclusion:**

Home-based isometric handgrip exercise using commercial spring devices at 20–50% MVC significantly reduced SBP in hypertensive older adults. It is a simple, safe, and affordable exercise option, suitable for those unable to perform standard aerobic activities.

## Introduction

Hypertension is highly prevalent in older adults and represents a major risk factor for cardiovascular and cerebrovascular diseases. In Thailand, the prevalence of hypertension continues to rise, with a significant proportion of individuals either undiagnosed or inadequately treated [1]. While aerobic exercise is a well-established non-pharmacological intervention [2], it is frequently underutilized in older populations due to physical limitations, comorbidities, or poor adherence [3–4].

Isometric resistance exercise, particularly isometric handgrip training (IHT), has emerged as a promising adjunct therapy for blood pressure control. Several randomized controlled trials and meta-analyses have demonstrated that IHT can significantly reduce blood pressure in a variety of populations, including normotensive, pre-hypertensive, and hypertensive adults [5–8]. The 2017 American Heart Association guidelines recommend an IHT protocol consisting of handgrip contractions at 30–40% of maximum voluntary contraction (MVC), held for two minutes per set across four sets, with one-minute rest intervals, three times per week over 8–10 weeks. This protocol has been associated with average reductions in systolic blood pressure of 4–5 mmHg [2]. While the Thai Hypertension Guideline emphasizes moderate-intensity aerobic exercise and does not include resistance or isometric training, [1] we were interested in examining the effects of IHT using the AHA-recommended protocol in older adults with hypertension.

However, most existing trials have been conducted in hospital or laboratory settings, using expensive handgrip dynamometers under direct supervision. This limits the practicality and accessibility of IHT, particularly for older adults who are unable to perform standard aerobic exercise. Despite its simplicity, short training duration, and potential to be incorporated into daily activities, few studies have evaluated its effectiveness as a home-based intervention—especially in the elderly population. This study aims to investigate whether isometric handgrip exercise, using an affordable commercial device, is a feasible and effective home-based strategy for blood pressure reduction in older adults with hypertension.

## Materials and methods

This assessor-blinded randomized controlled trial was conducted from September 2020 to June 2021 in accordance with the principles of the Declaration of Helsinki [9], approved by the Institutional Review Board of the Faculty of Medicine, Chulalongkorn University (IRB No. 419/63) and registered with the Thai Clinical Trials Registry (TCTR20200817003). Participants were recruited from the Comprehensive Geriatric Clinic, King Chulalongkorn Memorial Hospital, Bangkok, Thailand. Eligible participants were identified through medical record reviews and referrals from internal medicine physicians and clinic nurses. Inclusion criteria were adults aged 60 years or older with a clinical diagnosis of hypertension, with or without medication, and a maximum grip strength between 10 and 40 kg. Exclusion criteria included uncontrolled hypertension (resting systolic blood pressure ≥160 mmHg or diastolic blood pressure ≥100 mmHg) [10], contraindications to resistance training [11], or musculoskeletal conditions limiting IHT (e.g., trigger finger, hand osteoarthritis, rheumatoid arthritis). Written informed consent was obtained from all participants prior to participation. Participants were randomly assigned to either the intervention or control group using a computer-generated block-of-four randomization method. The random sequence was created by an investigator who was not involved in participant recruitment, intervention delivery, or data analysis. Allocation was concealed using sequentially numbered, opaque, sealed envelopes that were opened only after baseline assessments were completed. Outcome measurements were performed by an assessor who was blinded [9] to group allocation. The sample size was calculated based on the mean systolic blood pressure difference and standard deviation reported by Millar PJ et al. [12], with a study power of 90% (β = 0.10), a significance level of 5% (α = 0.05), and an estimated dropout rate of 30%. The required sample size was 32 participants, with 16 in each group.

### IHT intervention

The intervention protocol was developed based on protocols in the recommendation from the 2017 American Heart Association hypertension guidelines, which suggest training intensities between 30% and 50% of MVC [2]. Due to the lack of commercially available handgrip devices with adjustable resistance, we adapted the protocol to be feasible and suitable for unsupervised home-based training.

Participants in the intervention group were stratified into two resistance levels—5 kg and 10 kg—based on commercially available spring-loaded handgrip devices (Handgrip 5 kg and 10 kg, Daiso, China) (S1 Fig).The devices were visually inspected for wear before and after the program and no change in resistance were reported. Each participant performed three MVC trials using their preferred hand with a JAMAR Plus+ digital hand dynamometer, and the average value was used to guide device assignment. Participants with an MVC of 10–19.9 kg were assigned the 5 kg device, while those with an MVC of 20–40 kg were assigned the 10 kg device, corresponding approximately to 25–50% MVC. The exercise protocol consisted of four 2-minute sets of sustained isometric handgrip contraction using the assigned device, with 1-minute rest intervals between sets. Exercises were performed with the participant’s preferred hand.

### Control group

Participants in the control group were instructed to perform non-resistive hand exercises with the same schedule as the intervention group. They were asked to mimic the gripping motion without applying force, completing four 2-minute sets with 1-minute rest intervals using their preferred hand.Upon study completion, control group participants were provided with a handgrip device corresponding to their MVC category and received a supervised training session for personal use.

### Exercise protocol and monitoring

The initial exercise session was conducted under researcher supervision to ensure safety, proper technique, and hemodynamic stability (SBP ≤ 220 mmHg, DBP ≤ 105 mmHg). Thereafter, participants performed the exercise independently at home three times per week for eight weeks. They received detailed instructions and an exercise diary to record each session. Biweekly phone calls were made to monitor adherence, check for adverse events, and confirm that no changes occurred in antihypertensive treatment or overall physical activity levels.

### Outcome measurements

The primary outcome was systolic blood pressure (SBP); secondary outcomes included diastolic blood pressure (DBP), heart rate (HR), grip strength, skeletal muscle mass (SMM), and exercise satisfaction. All measurements were performed by a blinded assessor at baseline and at 8 weeks.

### Blood pressure and heart rate

Participants abstained from alcohol, caffeine, smoking, and exercise for at least 30 minutes prior to measurement. After voiding and resting for 10 minutes in a quiet, temperature-controlled room, BP and HR were recorded using an automated device (Microlife BP A200 AFIB) with a proper cuff-size to arm-circumference ratio. Three readings were taken at 2-minute intervals, and the average was used. Measurements were conducted at approximately the same time of day of both visits to minimize diurnal variation and antihypertensive medication cycles [2].

### Grip strength

Grip strength was measured using the Jamar Plus+ digital dynamometer in seated position, with the shoulder adducted, elbow flexed at 90° and wrist in neutral position. Three maximal voluntary contractions were performed at 1-minute intervals, and the mean value was used for analysis [13].

### Skeletal Muscle Mass (SMM)

SMM was measured using the InBody 770 (Inbody, Seoul, Korea), a validated bioelectrical impedance analyzer routinely used in the clinical setting, and recommended in sarcopenia guideline [14].

### Physical activity and satisfaction

The Thai version of the short-form International Physical Activity Questionnaire (IPAQ) [15] was administered at both visits to track activity changes. At 8 weeks, participants completed a satisfaction questionnaire using a Likert scale and provided open-ended feedback on exercise experience and any adverse effects.

### Statistical Analyses

The data was analyzed using SPSS Statistics Version 22 (IBM Corp., Armonk, NY, USA). Descriptive data were summarized as mean and standard deviation (SD) or percentages, as appropriate. Within-group comparisons were analyzed using paired t-tests. Between-group differences were analyzed using analysis of covariance (ANCOVA), adjusting for baseline values. Both per-protocol and intention-to-treat (ITT) analyses were conducted, yielding comparable results; therefore, ITT results are reported. Missing data were handled using the last observation carried forward (LOCF) method. All statistical tests were two-tailed, with a significance level set at *p* < 0.05.

### Results

**Fig 1:**
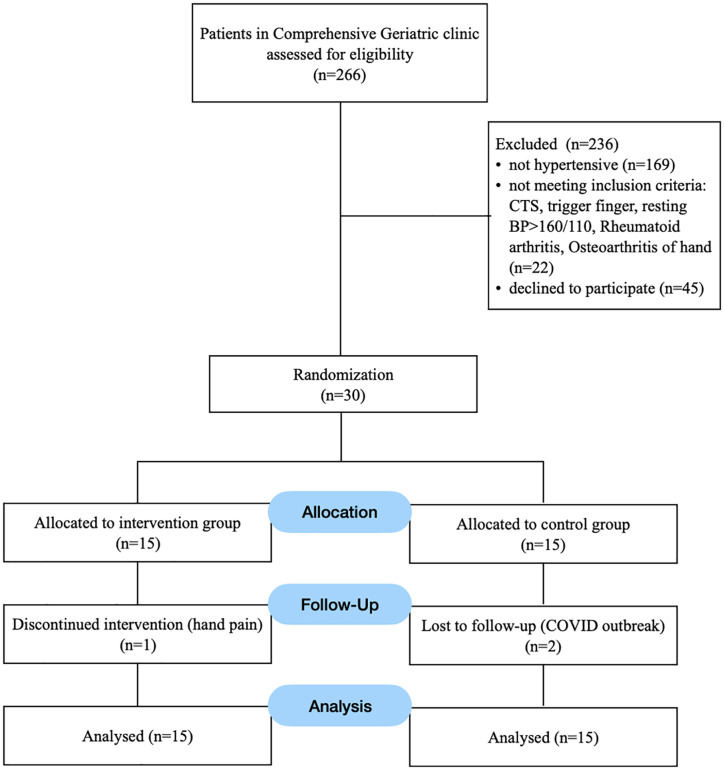
Flow of participants.

A total of 30 participants were enrolled, with 3 dropouts—1 in the intervention group due to transient hand pain, and 2 in the control group due to transportation issues during the COVID-19 outbreak. Fig 1 shows participants flow diagram. Baseline characteristics were comparable between groups (Table 1).

**Table 1:** Baseline characteristic of participants.

	Intervention (n = 15)	Control (n = 15)	p-value
Age (y)	69.4 ± 4.7	69.9 ± 6.6	0.705
Female	9 (60)	10 (66.7)	0.351
Weight (kg)	63.6 ± 9.0	63.7 ± 15.6	> 0.99
BMI (kg/m^2^)	25.1 ± 2.1	25.8 ± 4.3	0.631
Dominant hand used for exercise	11 (73.3)	8 (53.3)	0.127
Comorbidities			> 0.99
Diabetes mellitus	3(20)	1 (6.7)	
Dyslipidemia	8 (53.3)	8 (53.3)	
Coronary artery disease	1 (6.7)	0	
Cerebrovascular disease	2 (13.3)	2 (13.3)	
Number of antihypertensive medications			0.484
None	1 (6.7)	0	
1	9 (60.0)	10 (66.7)	
2	4 (26.7)	2 (20)	
3	1 (6.7)	2 (13.3)	
Short IPAQ: inactive	9 (60.0)	7 (46.7)	0.273
SBP (mmHg)	139.1 ± 12.9	141.2 ± 9.7	0.618
DBP (mmHg)	79.1 ± 10.2	82.9 ± 7.9	0.263
HR (BPM)	73.0 ± 10.4	69.4 ± 12.7	0.287
Hand grip strength (kg)	25.9 ± 8.3	23.7 ± 6.7	0.431
SMM (kg)	22.9 ± 5.2	21.6 ± 5.6	0.515

Values are presented as mean ± standard deviation or number (%)

BMI, body mass index; IPAQ, International physical activity questionnaire; SBP, systolic blood pressure; DBP, diastolic blood pressure; BPM, beat per minute; SMM, skeletal muscle mas

**Fig 2:**
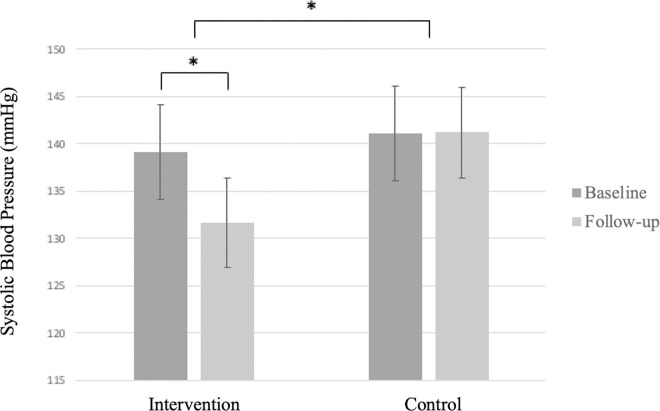
Baseline and 8-week systolic blood pressure (SBP) in the intervention and control groups. Bars represent mean SBP (mmHg) ± standard deviation, * indicates *p* < 0.05.

**Fig 3:**
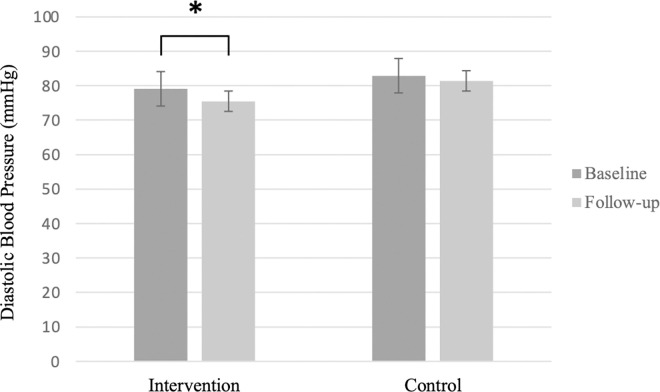
Baseline and 8-week diastolic blood pressure (DBP) in the intervention and control groups. Bars represent mean SBP (mmHg) ± standard deviation,* indicates *p* < 0.05.

In the intervention group, participants received handgrip devices corresponding to their MVC levels, averaging 34.39 ± 6.26% MVC; 4 received 5 kg and 11 received 10 kg devices. After 8 weeks, the intervention group showed significant reductions in SBP and DBP, while the control group showed no significant change in SBP or DBP (Figs 2 and 3). Grip strength improved significantly in both groups, with no significant change in HR or SMM (Table 2). Between-group analysis showed a significant difference in SBP reduction, but no significant differences in other outcomes (Table 3). Exercise compliance was high in both groups, with more than 95% of prescribed sessions completed. Most participants in the intervention group reported high satisfaction and willingness to continue, citing the simplicity and convenience of the exercise. Three participants experienced transient hand or forearm pain during early sessions, all of which resolved without intervention. No serious adverse events or musculoskeletal complications were reported.

**Table 2:** Within-group comparison between baseline and follow-up at 8-week (N = 15 per group).

	Intervention (N =15)			Control group (N = 15)		
	Baseline	Follow-up	p-value	Baseline	Follow-up	p-value
SBP (mmHg)	139.1 ± 12.8	131.7 ± 8.7	0.011*	141.2 ± 9.7	141.1 ± 13.8	0.983
DBP (mmHg)	79.1 ± 10.2	75.5 ± 8.2	0.030*	82.9 ± 7.9	81.4 ± 6.2	0.374
HR (BPM)	73.0 ± 10.4	75.5 ± 15.1	0.470	69.4 ± 12.7	71.8 ± 8.2	0.350
Grip strength (Kg)	25.9 ± 8.3	27.5 ± 7.8	0.003*	23.7 ± 6.7	24.8 ± 7.8	0.033*
SMM (Kg)	22.9 ± 5.1	22.8 ± 5.0	0.454	21.6 ± 5.9	22.1 ± 5.7	0.126

Values are presented as mean ± standard deviation

SBP, systolic blood pressure; DBP, diastolic blood pressure, BPM, beat per minute; SMM, skeletal muscle mass

*p < 0.05

**Table 3:** Between-group comparison of mean changes from baseline and follow-up (N = 15 per group).

	Intervention (N = 15)	Control group (N = 15)	
	Mean change (95%CI)	Mean change (95%CI)	P value
SBP (mmHg)	− 7.3 (−12.2 to −2.4)	− 0.1 (−6.1 to 5.9)	0.03*
DBP (mmHg)	− 3.6 (−6.6 to −0.6)	− 1.5 (−4.8 to 1.8)	0.06
HR (BPM)	2.5 (−4.2 to 9.2)	2.4 (−2.4 to 7.2)	0.66
Grip strength (Kg.)	1.6 (0.7 to 2.5)	1.1 (0.2 to 2.0)	0.50
SMM (Kg.)	−0.1(−0.4 to 0.2)	0.5 (−0.1 to 1.1)	0.11

Values are presented as mean ± standard deviation

SBP, systolic blood pressure; DBP, diastolic blood pressure, BPM, beat per minute; SMM, skeletal muscle mass

*p < 0.05

## Discussion

This study demonstrates that a home-based IHT protocol, using inexpensive, commercially available devices, significantly reduces SBP in older adults with hypertension. The intervention group achieved a mean SBP reduction of 7.33 mmHg after 8 weeks, which is consistent with previous findings. Millar et al. reported a between-group systolic blood pressure reduction of 9 mmHg in normotensive participants using an inexpensive spring handgrip device, corresponding to an effect size of Cohen’s d ≈ 3.2 [12]. Almeida et al. reported a similar SBP reduction of 8.11 mmHg in hypertensive patients undergoing IHT training, particularly those on stable antihypertensive therapy [16]. Similarly, a recent trial found that 8 weeks of IHT reduced office SBP by 8 mmHg, although no changes were observed in ambulatory blood pressure [17]. While the magnitude of SBP reduction observed in this study is smaller than the average effect of pharmacologic therapy which could lower SBP up to approximately 10 mmHg [18], it remains clinically meaningful. A meta-analysis of 344,716 participants from 48 randomized trials showed that a reduction of 5 mmHg in office SBP was associated with a 10% lower risk of major cardiovascular events, even among individuals with a mean age of 65 years [19]. Therefore, IHT may offer valuable adjunctive benefits, particularly for older adults who face physical limitations that prevent them from engaging in aerobic exercise.

However, no participant in our study achieved a post-intervention SBP < 120 mmHg—a target associated with reduced cardiovascular risk in the SPRINT trial—highlighting the challenge of intensive BP control in this population [20]. Guidelines differ on optimal BP targets: the 2017 ACC/AHA recommends a target of <130/80 mmHg for older adults [2], while the 2018 ESC/ESH suggests <140/90 mmHg [21], underscoring the need for individualized treatment based on frailty and comorbidities [22].

Although IHT may provide supplemental benefit, it should not replace aerobic exercise in individuals capable of performing it. A prior randomized trial found that only aerobic exercise, not IHT or sham handgrip training, led to significant reductions in ambulatory BP [23]. Moreover, a recent meta-analysis indicated that IHT reduces awake diastolic BP but has no significant effect on 24-hour, asleep, or awake systolic BP, further suggesting a limited role for IHT in modifying ambulatory BP [24].

In our study, both groups demonstrated improvements in grip strength, which could partly result from a learning effect form repeated testing; however, only the intervention group showed a significant reduction in blood pressure. This finding suggests that the antihypertensive effect of IHT is not directly attributable to gains in muscular strength alone. Although the exact mechanisms remain unclear, current evidence suggests that reductions in total peripheral resistance may be involved [25]. Rodrigues et al. reported that 12 weeks of IHT led to improvements in arterial stiffness and markers of endothelial function, despite no significant changes in inflammatory or oxidative stress markers [26]. Similarly, Li et al. reported that IHT significantly improved flow-mediated dilation (FMD) compared with controls, indicating enhanced endothelial function. However, all FMD assessments in their analysis were performed on the brachial artery, which corresponds anatomically to the primary site of forearm muscle contraction. This proximity may accentuate local vascular adaptations [27]. Although the present study involved unilateral training of the dominant hand and blood pressure was measured on the same side, previous research indicates that IHT can still elicit systemic blood pressure reductions through central autonomic mechanisms. Maier et al. conducted a systematic review and meta-analysis demonstrating that muscle sympathetic nerve activity consistently increases during isometric handgrip exercise and remains elevated during post-exercise circulatory occlusion, supporting the role of the metaboreflex independent of the exercise pressor reflex [28]. These findings suggest potential central adaptations that may contribute to the systemic blood pressure reduction observed following IHT. Nevertheless, further longitudinal studies are needed to clarify whether these neural and vascular adaptations translate into sustained structural vascular remodeling [23].

To our knowledge, this is the first study to implement IHT using an affordable, fixed-resistance device (~60 THB) instead of expensive specialized equipment. The home-based approach makes it more accessible for older adults who may face barriers to participating in hospital-based programs [29], thus enhancing its real-world applicability, particularly in resource-limited settings.

This study has several limitations. First, blood pressure was measured in the office, which may not be as accurate as home or ambulatory blood pressure monitoring. Second, blood pressure was assessed in the same limb used for the exercise, limiting our ability to assess the systemic effects of IHT on blood pressure. Third, the relatively small sample size and feasibility nature of the study, along with the missing data handled using the LOCF method, may limit the generalizability of the results to larger or more diverse populations. Finally, the study lacked long-term follow-up, so the sustainability of the hypotensive effects of IHT remains unknown.

Future research should involve larger-scale trials with more diverse populations and include long-term follow-up to assess the lasting effects of IHT on blood pressure. Incorporating more accurate blood pressure measurement methods, such as 24-hour ambulatory blood pressure monitoring, along with cardiovascular biomarkers, may provide a deeper understanding of the systemic benefits and potential mechanisms of IHT.

## Conclusion

The home-based isometric handgrip exercise, using commercially available devices, resulted in a significant reduction in SBP by 7.33 mmHg for older adults with hypertension, with a meaningful difference between the intervention and control groups. However, no statistically significant differences were observed in other outcomes.

## Supporting information

S1 Fig Spring-loaded handgrip devices used for the home-based intervention. (TIFF)

S1 Checklist CONSORT 2010 checklist of information to include when reporting a randomized trial. (DOCX)

S1 Protocol Research protocol as approved by the Institutional Review Board. (DOCX)
