# Supplementary figures and images for: Effect of home-based isometric handgrip exercise with a commercially available device on blood pressure in older adults with hypertension: A randomized controlled trial
Source: PLoS One. 2026 Mar 4;21(3):e0342563. doi: 10.1371/journal.pone.0342563 (PMC12959700; doi:10.1371/journal.pone.0342563)

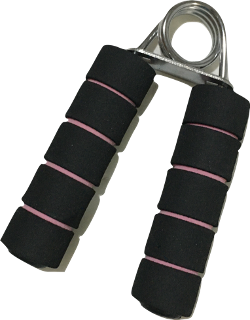

Supplement: S1 Fig — (TIFF) [file pone.0342563.s001.tiff]
