# Supplementary material for: Effect of home-based isometric handgrip exercise with a commercially available device on blood pressure in older adults with hypertension: A randomized controlled trial
Source: PLoS One. 2026 Mar 4;21(3):e0342563. doi: 10.1371/journal.pone.0342563 (PMC12959700; doi:10.1371/journal.pone.0342563)
Supplement: S1 Protocol — (DOCX) [file pone.0342563.s003.docx]

**Research proposal**

**Proposal Title**

Effect of home-based isometric handgrip exercise with commercially available device on blood pressure in hypertensive older adults: a randomized controlled trial

ผลของการออกกำลังกายที่บ้านด้วยการบีบมือแบบเกร็งค้างโดยใช้อุปกรณ์ที่มีวางจำหน่ายทั่วไปต่อความดันโลหิตในผู้สูงอายุที่เป็นโรคความดันโลหิตสูง

**Investigators**

Primary investigator

Tanya Jitkaew, M.D.

Physical Medicine and Rehabilitation Resident

Co-investigators and advisors

Jirapa Champaiboon, M.D.

Sarissa Rangkla, Asst. Prof., M.D.

Aisawan Petchlorlian, M.D.

**Introduction**

Hypertension is a common disease among population especially in the elderly and is a major risk factor for developing cerebrovascular and cardiovascular disease. Prevalence of hypertension in Thailand has been increasing over time; about 24.7% of population was diagnosed with hypertension in 2013. Major problems are unawareness and uncontrolled blood pressure^(1)^. Despite pharmacological management, one of the best-proven non-pharmacological interventions for prevention and treatment of hypertension is physical activity or exercise^(2)^. The 90-150 minutes per week of moderate intensity aerobic exercise has been long known and often prescribed to patients^(2)^. However, aerobic exercise usually gains more popularity and adherence from young adult group rather than elderly group. The barriers to aerobic exercise training are mindset among many elderly that exercise is only for young adult, and multiple comorbidities in which the most common are arthritis and other musculoskeletal disorder, that interfere with walking and related activities^(3)^. It is often too hard and too difficult for most of the elderly hypertensive patients to perform aerobic exercise due to declined overall activity performance and frailty. There was a survey in 2015, reveals that among overall sedentary Thai population, about 43.6% are aged more than 50 years old. While among Thai population who have adequate physical activity, the elderly are counted for only 33.8%^(4)^. The challenge for clinicians is to convince the elderly, who usually have not been exercise for many decades, to start engaging in suitable physical activities and exercise for their age and physical function.

In recent years, isometric resistive exercise is known to be an alternative exercise to reduce blood pressure. Several randomized-controlled trials (RCT) have proven the effectiveness of this kind of exercise, systematic reviews and meta-analysis were also conducted and confirmed that isometric resistive exercise produces a significant anti-hypertensive effect in young normotensive adults, older pre-hypertensive, and older medicated hypertensive patients ^(5-13)^. The most common isometric resistive exercise training among several studies is isometric handgrip exercise. The training protocol usually use the 30% maximum voluntary contraction (MCV) as the intervention group and 5% MCV or sedentary as the control group, performing four set of 2 minutes isometric contraction with a brief rest, conducting period from 4-20 weeks^(5-7, 14)^.

A 2017 ACC/AHA Guideline for the Prevention, Detection, Evaluation, and Management of High Blood Pressure in Adults recommends 4 sets of 2-minutes of 30-40% maximum voluntary handgrip contraction with 1 minute resting between exercises, 3 sessions per week for 8-10 weeks, approximately reduces systolic blood pressure reduction by 5mmHg in hypertensive and 4mmHg in normotensive patients^(2)^.

Mechanism for explaining blood pressure reduction remains unclear, but several hypotheses are proposed. A reactive hyperemia due to isometric resistive exercise contributes to the increased basal production of nitric oxide that improved resistance vessel endothelial function^(9)^. The improved systemic endothelial function may leads to a reduction in systemic total peripheral resistance^(8)^. Other possible mechanisms include the autonomic modulation by decreased sympathetic, and enhanced parasympathetic modulation of both heart rate and blood pressure ^(9, 10)^, the increased training limb artery diameter, blood velocity, and blood flow in concert with reduced vascular conductance and enhanced endothelial-dependent vasodilatation^(11)^.

Although isometric handgrip exercise has been previously feared for increasing blood pressure during the resistive phase, many studies are conducted and proved that it is a safe, well-tolerated procedure and cause no adverse effects that induce harmful hemodynamic responses^(15, 16)^. A study in 2011 by Araújo et al investigated the acute response during acute isometric handgrip exercise in elderly populations. There was an increase in SBP, DBP and HR by 16mmHg, 7 mmHg and 3 bpm respectively^(16)^, and these effect had returned to baseline after 1 minute of post-exercise recovery. There was also a study conducting submaximal isometric handgrip training in active cardiopulmonary rehabilitation patients and reported to be well tolerated and requiring minimal exertion.^(17)^ Another worrisome effect is the post-exercise hypotension (PEH) that was commonly reported in aerobic exercise, a study in 2013 by Olher et al conducted in hypertensive elderly women reported that was no post-exercise hypotension or cardiovascular overload occurred in the isometric handgrip exercise.^(15)^

Despite promising evidences of isometric handgrip exercise on blood pressure reduction, most of the trials are hospital-based or lab-based training protocols under direct supervision using expensive dynamometer (20,000 THB) as exercise equipment.

In order to make isometric handgrip exercise becomes more practical and accessible, so that general population, especially in elderly patients who cannot tolerate or unable to perform standard aerobic exercise due to many difficulty and frailty, would gain benefits from practicing this at home without having to travel to lab or hospital to do the isometric handgrip exercise. Since isometric handgrip exercise is easy to perform, short duration of training, and could be perform along with their leisure activities and transportation. We want to test whether isometric resistive exercise is an effective home-based practical procedure. The aim of this study is to access the effectiveness of home-based isometric handgrip exercise using inexpensive commercial available device in reducing resting SBP in hypertensive elderly patients. We also would like to access the changes in resting DBP, HR and handgrip strength after being trained, together with the satisfaction and any possible musculoskeletal side effect of this exercise.

**Literature review**

In 2014, an anchor systematic review and meta-analysis by Debra J. Carlson et al has revealed the effectiveness of isometric resistance training lasting 4 or more weeks in lowering systolic blood pressure (SBP) by -6.77mmHg(95%CI -7.93,-5.62;P<0.001), diastolic blood pressure (DBP) by -3.96mmHg(95%CI -4.80,-3.12;P<0.001), and mean arterial pressure (MAP) by -3.94mmHg(95%CI -4.73,-3.16;P<0.001), respectively^(5)^. Another systematic review and meta-analysis in 2015 by Jodie D Inder et al reported a subgroup analysis that anti-hypertensive effects is larger in hypertensive male aged ≥45years, using unilateral arm isometric resistive training conducting for at least 8 weeks^(6)^. These two systematic reviews, which were cited in 2017 AHA’s hypertension guideline, retrieved data from common RCTs regarding isometric resistance exercise with different characteristics: unilateral/bilateral/alternating isometric handgrip contraction, double leg extension exercise.

In 2018, K.F. Goessler et al conducted a home-based randomized controlled trial using telemonitoring and telecoaching to compare BP response in isometric handgrip exercise versus moderate intensity aerobic exercise in healthy adults. Trial was 8 weeks duration with handgrip group performing daily, while aerobic group performing moderate intensity aerobic exercise for at least 150 minutes per week. The result showed a significant reduction in both ambulatory and office DBP in aerobic group, but significant reduction in only office SBP in handgrip group. They concluded that aerobic exercise should remain a number one recommendation in the prevention and treatment of hypertension, while isometric handgrip exercise could be a useful tool for lowing blood pressure in elderly adult or patients who have multiple comorbidities.^(18)^

There was a RCT in 2002 conducting an isometric handgrip exercise in hypertensive elderly population, aging 60-80 years old, and revealed significant hypotensive effect following 10 weeks of standard 4sets of 2minutes handgrip exercise at 30% MVC, 3 days per week. SBP was reduced from 156±9.4 to 137±7.8 mmHg in exercise group versus 152±7.8 to 144±11.8 mmHg in non-exercise group. 75% of the hypertensive elderly participants had been taking antihypertensive medication for many years.^(10)^

There was a RCT in 2008 using an inexpensive spring handgrip-training device with intensity about 30-40% MCV in normotensive patients, also demonstrated a significant reduction in resting SBP and DBP, 10±3 mm Hg and 3±1 mm Hg respectively. The study design was 3 sessions/week, training in lab-based 2 sessions/week and home-based 1 session/week, and was carried on for 8 weeks. They used 3 different intensity handgrip spring device based on each MVC. The control group was arranged for discussion session about hypertension once a week. ^(13)^

**Study design**

This is a randomized-controlled single blinded trial. The assessors are blinded from knowing which group participants are assigned. Eligible participants are randomized into either intervention group or control group using blocked randomization.

**Method**

This study will be conducted after being approved by the Institutional Review Board of the Faculty of Medicine, Chulalongkorn University, Bangkok, Thailand.

**Participants**

Participants are recruited from Comprehensive Geriatric Clinic in King Chulalongkorn Memorial Hospital, Bangkok, Thailand. The doctor and nurse in clinic are contacted and informed by researchers about this project and refer eligible population to meet researcher. Referring physician will be masked from knowing if the patients decide to join the study or not.

**Inclusion criteria.**

1. Elderly adults, aged 60 years or more.
2. Diagnosed and being treated for hypertension, whether receiving pharmacotherapy or not.
3. Maximum grip strength ranging from 10-40kg.
4. Willing to join the study.

**Exclusion criteria.**

1. Resting SBP≥160 or DBP≥100^(19)^.
2. Contraindicated for resistance exercise^(20)^:
   - Recent myocardial infarction / CABG within 12weeks
   - Arrhythmia
   - Acute congestive heart failure
   - Unstable angina
   - Severe joint or muscle pain during unresisted movement
   - Inflammatory neuromuscular disease
3. Musculoskeletal problems preventing handgrip exercise such as

- Trigger finger

- Carpal tunnel syndrome

- De Quervain’s tenosynovitis

- Rheumatoid arthritis

- Osteoarthritis of hand

**Dropout criteria** Patients with one or more of the following conditions are classified as dropout

- Antihypertensive medication adjustment within study period.
- Physical activity or exercise adjustment within study period.
- Patients who have complication regarding handgrip exercise.

**Procedure**

Patients are screened for eligibility and provided information about this study by researcher. They are free to make decision in participating the study. The willing patients then signed an informed consent for participation. They are asked to choose their preferred hand for training and use that side for training and measurement.

**Isometric Handgrip Exercise intervention group**

We have reviewed the commonly used protocol in previous studies and 2017 AHA’s hypertension guideline; intensity in the intervention group is usually 30%-50%MVC. Since there is no commercially available spring handgrip device that can precisely adjust resistance load to the desirable value, we make an adaptation to protocol to make it more simple and suitable for home-based exercise. We classify participants into 2 intensity levels: 5kg and 10kg resistance load, based on commercially available device.

All participants are asked to complete 3 maximum voluntary contractions (MVC) in preferred hand using Jamar plus+ digital hand dynamometer to determine their average MVC. Participants in the intervention group with MVC range from 10-19.9kg or 20-40kg are provided a 5kg or 10 kg resistance load handgrip device respectively, which approximated their 25%-50% MVC. They use the provided handgrip device to complete 4 sets of 2-minutes handgrip contraction with 1 minute resting between exercises, with chosen hand.

**Control group**

Participants are asked to do non-resistive hand exercise with the chosen hand by simply grasp without squeezing, in total of 4 sets of 2-minutes grabbing with 1 minute resting between exercises.

After completing the study, the spring handgrip device with corresponding resistance level will be given to each participants in control group, also with exercise training session from researcher.

**Both groups**

First exercise session is conducted under supervision by a researcher. The immediate hemodynamic effect of exercise is obtained during this session to ensure that the increased blood pressure during resistive exercise is within safety limit of SBP≤220 and DBP≤105^(19)^. All participants are required to familiarize themselves with their study protocol before leaving their first visit at clinic and exercise diary is given.

During 8 weeks of study, both groups are committed to perform their procedure 3 days per week and record their activity in exercise diaries that were given. A telephone visit by research member is scheduled every 2 weeks to surveillance for adherence to the study protocol, complication and to ensure no changes in antihypertensive medications or exercise.

**Outcome measurement**

Baseline blood pressure, grip strength and skeleton muscle mass (SMM) are measured at the first visit. After 8 weeks, all participants are appointed at clinic to assess blood pressure, grip strength and SMM. We use InBody 770 body composition and body water analyzer to measure SMM. Thai short IPAQ is used to assess the physical activity level at both visits^(21)^. Questionnaire is given concerning the satisfaction of this exercise and any possible post-procedural musculoskeletal side effect. Blood pressure, grip strength and SMM are measured by the same research member who is blinded to randomization and treatment procedure.

**Blood pressure measurement**

Following the blood pressure measurement protocol in 2017 AHA’s hypertension guideline, participants are required to abstain from alcohol, caffeine, smoking and exercise for at least 30 minutes before measurement. Blood pressure and heart rate are measured in a seated position following bladder voiding and 10 minutes of resting in quiet, temperature-controlled room^(2)^. We use Nissei DM-3000 automated digital blood pressure monitor with appropriate cuff size placed on the arm they have chosen for procedure. They are measured for 3 times with 2 minutes interval between each measurement and the average value is used for analysis. To control diurnal variation of blood pressure and medication intake cycle, measurement at baseline and post-procedure are done approximately at the same time of day. The time of ingesting antihypertensive medication is standardized.

**Grip strength measurement**

Grip strength measurement is conducted in the same position as exercise position. Subjects held Jamar plus+ digital hand dynamometer with their preferred hand and conduct a maximum voluntary contraction in seated position, shoulders adducted and neutrally rotated, elbow flexed at 90°, forearm in neutral and wrist between 0 and 30° of dorsiflexion. Three trials are taken at 1min intervals. The mean of three trials is used for analysis^(21)^.

**Questionnaire**

Satisfaction of the exercise is rated in Likert scale, providing quantitative data. Questions asking if participants would continue to perform this exercise after the study, any comments and complications regarding exercise are pursued in descriptive data.

**Sample size calculation**

A sample size of 36 participants (18 patients per group) is calculated based on mean difference and SD from Millar PJ et al. study in 2008^(13)^ with 5% error and 90% power.

Formular n/group = 2(Zα/2 + Zβ)2 σ2 / (X1-X2)2

where σ2 = [(n1-1) S12+(n2-1)S22] / (n1+ n2 - 2)]

X1-X2 = (-10) – (-1) = 9, SD1 = 8.98, SD2 = 2.36, n1 = 24, n2 = 25

α = 0.05, β = 0.10, Zα/2 = 1.96. Zβ = 0.84

n/group ~ 12

Since home-based protocol is susceptible to higher dropout rate, we estimate 30% of dropout rate and calculate total n/group = 18

**Statistical analysis**

Statistical analyses are performed using SPSS Statistics Version 22. Demographic data are analyzed and present as mean, SD and percent. Within group comparison is used to analyze pre-post treatment using paired T-test. Between-group comparison is used to analyze mean difference between group using unpaired T-test if baseline characteristics are similar, or ANCOVA if baseline characteristics are different. All statistical tests are two-tailed, statistical significance is set at p<0.05, and using intention to treat analysis.

**Feasibility of protocol**

Base on year 2019, number of elderly patients in Comprehensive Geriatrics Clinic at King Chulalongkorn Memorial Hospital is about 100 patients per month. Total sample size that planned to be enrolled in this study is 36. So, It has a high chance of being successfully completed.

**Ethical consideration**

Respect of person: All participants enrolled in this study will be well informed regarding the methods, aims and possible side effects of treatments. All participants will voluntarily provide their informed consent without feeling any pressure and can withdraw at any time. All information provided from participants is treated as confidential and will not be released by the researcher to a third party.

Beneficence/Non-maleficence: Isometric handgrip exercise is reported to be an alternative and effective exercise in reducing blood pressure. No serious side effects or complications from isometric handgrip exercise was reported in the literature. Although researcher will try to protect participants from any harm and treat if it occurs.

Justice: This study has clear inclusion and exclusion criteria for participation.

**Expected benefits gain**

If our protocol for home-based isometric exercise using commercial available device could result in blood pressure reduction in hypertensive elderly adults, it would be an alternative or add-on exercise that can help in blood pressure management in patients who are unable to perform aerobic exercise.

**Timeline**

Jan-May 2020 formulate the study protocol and applying for funding from grants for research and approval of Institutional Review Board of the Faculty of Medicine, Chulalongkorn University

June 2020 – June 2021 start recruiting participant after being approved and conduct the study

July-Dec 2021 analysis and conclusion of the study

**Keywords**

Isometric handgrip exercise, isometric resistance training, grip strength, home-based, hypertension

**Reference:** all data are retrieved on 4 January 2020

1. แนวทางการรักษาความดันโลหิตสูงในเวชปฏิบัติทั่วไป พ.ศ.2562. สมาคมความดันโลหิตสูงแห่งประเทศไทย: ทริค ธิงค์, 2562.

2. Whelton PK, Carey RM, Aronow WS, Casey DE, Jr., Collins KJ, Dennison Himmelfarb C, et al. 2017 ACC/AHA/AAPA/ABC/ACPM/AGS/APhA/ASH/ASPC/NMA/PCNA Guideline for the Prevention, Detection, Evaluation, and Management of High Blood Pressure in Adults: A Report of the American College of Cardiology/American Heart Association Task Force on Clinical Practice Guidelines. Hypertension. 2018;71:e13-e115.

3. Fleg JL. Aerobic exercise in the elderly: a key to successful aging. Discov Med. 2012;13:223-8.

4. Liangruenrom N, Topothai T, Topothai C, Suriyawongpaisan W, Limwattananon SL, C., al e. Do Thai People Meet Recommended Physical Activity Level?: The 2015 National Health and Welfare Survey. Journal of Health Systems Research 2015;2:205-20.

5. Carlson DJ, Dieberg G, Hess NC, Millar PJ, Smart NA. Isometric exercise training for blood pressure management: a systematic review and meta-analysis. Mayo Clin Proc. 2014;89:327-34.

6. Inder JD, Carlson DJ, Dieberg G, McFarlane JR, Hess NC, Smart NA. Isometric exercise training for blood pressure management: a systematic review and meta-analysis to optimize benefit. Hypertens Res. 2016;39:88-94.

7. Carlson DJ, Inder J, Palanisamy SK, McFarlane JR, Dieberg G, Smart NA. The efficacy of isometric resistance training utilizing handgrip exercise for blood pressure management: A randomized trial. Medicine (Baltimore). 2016;95:e5791.

8. McGowan CL, Visocchi A, Faulkner M, Verduyn R, Rakobowchuk M, Levy AS, et al. Isometric handgrip training improves local flow-mediated dilation in medicated hypertensives. European Journal of Applied Physiology. 2006;99:227-34.

9. Badrov MB, Bartol CL, DiBartolomeo MA, Millar PJ, McNevin NH, McGowan CL. Effects of isometric handgrip training dose on resting blood pressure and resistance vessel endothelial function in normotensive women. Eur J Appl Physiol. 2013;113:2091-100.

10. Taylor AC, McCartney N, Kamath MV, Wiley RL. Isometric training lowers resting blood pressure and modulates autonomic control. Med Sci Sports Exerc. 2003;35:251-6.

11. McGowan CL, Levy AS, Millar PJ, Guzman JC, Morillo CA, McCartney N, et al. Acute vascular responses to isometric handgrip exercise and effects of training in persons medicated for hypertension. Am J Physiol Heart Circ Physiol. 2006;291:H1797-802.

12. Bentley DC, Thomas SG. Characterizing and Comparing Acute Responses of Blood Pressure, Heart Rate, and Forearm Blood Flow to 2 Handgrip Protocols. J Cardiopulm Rehabil Prev. 2018;38:400-5.

13. Millar PJ, Bray SR, MacDonald MJ, McCartney N. The hypotensive effects of isometric handgrip training using an inexpensive spring handgrip training device. J Cardiopulm Rehabil Prev. 2008;28:203-7.

14. Jorgensen MG, Ryg J, Danielsen MB, Madeleine P, Andersen S. Twenty weeks of isometric handgrip home training to lower blood pressure in hypertensive older adults: a study protocol for a randomized controlled trial. Trials. 2018;19:97.

15. Olher Rdos R, Bocalini DS, Bacurau RF, Rodriguez D, Figueira A, Jr., Pontes FL, Jr., et al. Isometric handgrip does not elicit cardiovascular overload or post-exercise hypotension in hypertensive older women. Clin Interv Aging. 2013;8:649-55.

16. Araujo CG, Duarte CV, Goncalves Fde A, Medeiros HB, Lemos FA, Gouvea AL. Hemodynamic responses to an isometric handgrip training protocol. Arq Bras Cardiol. 2011;97:413-9.

17. Gordon BDH, Whitmire S, Zacherle EW, Doyle S, Gulati S, Leamy LJ, et al. “Get a Grip on Hypertension”. Journal of Cardiopulmonary Rehabilitation and Prevention. 2019;39:E31-E4.

18. Goessler KF, Buys R, VanderTrappen D, Vanhumbeeck L, Cornelissen VA. A randomized controlled trial comparing home-based isometric handgrip exercise versus endurance training for blood pressure management. J Am Soc Hypertens. 2018;12:285-93.

19. American College of Sport Medicine. ACSM’s guidelines for exercise testing and prescription. 10th ed. Philadelphia: Wolters Kluwer Health; 2018.

20. Carolyn Kisner LA. Therapeutic exercise: foundation and techniques. 5th ed. Philadelphia: F.A. Davis Company; 2007.

21. Rattanawiwatpong P, Khunphasee A, Pongurgsorn C, Intarakamhang P. Validity and reliability of Thai version of short format international physical activity questionnaire (IPAQ). J Thai Rehabil. 2006;16(3):147-60.

22. Roberts HC, Denison HJ, Martin HJ, Patel HP, Syddall H, Cooper C, et al. A review of the measurement of grip strength in clinical and epidemiological studies: towards a standardised approach. Age Ageing. 2011;40:423-9.
